# Terpenoid Esters Are the Major Constituents From Leaf Lipid Droplets of *Camellia sinensis*

**DOI:** 10.3389/fpls.2019.00179

**Published:** 2019-02-26

**Authors:** Xin Zhou, Xiaobing Chen, Zhenghua Du, Yi Zhang, Wenjing Zhang, Xiangrui Kong, Jay J. Thelen, Changsong Chen, Mingjie Chen

**Affiliations:** ^1^College of Horticulture and Fujian Provincial Key Laboratory of Haixia Applied Plant System Biology, Fujian Agriculture and Forestry University, Fujian, China; ^2^Tea Research Institute, Fujian Academy of Agricultural Sciences, Fujian, China; ^3^FAFU-UCR Joint Center for Horticultural Plant Biology and Metabolomics, Haixia Institute of Science and Technology, Fujian Agriculture and Forestry University, Fujian, China; ^4^Division of Biochemistry, Interdisciplinary Plant Group, Christopher S. Bond Life Science Center, University of Missouri, Columbia, MO, United States

**Keywords:** *Camellia sinensis*, lipid droplets, neutral lipids, sterol esters, triterpenoid esters, *CsPSAT1*, *CsHMGRs*, *CsAST1*

## Abstract

Lipid droplets (LDs) have been widely found from diverse species and exhibit diverse functions. It remains unexplored what potential roles they played in tea. To address this question, we analyzed the chemical composition and the dynamic changes of cytosolic LDs during leaf growth and diurnal cycle. Using TopFluor cholesterol and Nile Red staining we demonstrated that cytosolic LDs were heterogeneous in tea tree (*Camellia sinensis* cv. *Tieguanyin*); the size and number of LDs increased with leaf growth. Compositional analysis showed that terpenoid esters and diacylglycerol are the major components of cytosolic LDs. The contents of total sterol esters (SEs) and β-amyrin esters increased with leaf expansion and growth; individual SE also showed diurnal changes. Our data suggest that cytosolic LDs from tea tree leave mainly serve as storage site for free sterols and triterpenoids in the form of esters. Cytosolic LDs were not the major contributors to the aroma quality of made tea.

## Introduction

Lipid droplets (LDs) are intracellular macromolecular assemblies of neutral lipid esters or lipid-based polymers that are bound by a phospholipid monolayer membrane plus a variety of lipid-associated proteins (Murphy, 2012). Although, LDs have been commonly found from different species including virus, bacteria, fungi, algae, plants and animals, their neutral lipid composition varies dramatically (Mills and Cantino, 1977; Dubinsky et al., 1978; Steinbuchel and Fuchtenbusch, 1998; Welter et al., 1998; Mclauchlan, 2000; Bi et al., 2016).

Majority of seeds store TAGs in the form of lipiddroplets (Tzen et al., 1993), a desert shrub *Jojoba chinensis* stores liquid wax instead of TAGs (Pollard et al., 1979). In canola, phytosterols constitute about 0.5% of seed oil (Sabir et al., 2003), a third of which is in the form of SEs (Harker et al., 2003). Lotus seed embryo is a rich source of SEs (10% of crude oil), sitosterol esters and Δ^5^-avenasterol esters were the dominant SEs. The elaioplasts of tapetum contain numerous small LDs consisting of SEs and TAGs (Hernández-Pinzón et al., 1999). The scutellum and endosperm of the maize seedling are also rich in SEs (Kemp and Mercer, 1968).

LDs are also widely found in nonseed LDs. Large quantity of TAGs is stored in Avocado mesocarp in the form of LDs (Horn et al., 2013). In contrast, a third of stored lipids from wheat leaf LDs are in the form of sterol or wax esters (Parker and Murphy, 1981). Sterol ester synthesis in plants is catalyzed by *Phospholipid Sterol Acyltransferase1* (*PSAT1*) (Banas et al., 2005; Bouvier-Navé et al., 2010). An alternative pathway is catalyzed by *sterol O-acyltransferase* (*AST1*) with cycloartenol as acyl acceptor and saturated fatty acyl-Coenzyme A as acyl donor (Chen et al., 2007). In potato, a knockout of *PSAT1* resulted in a reduced number of leaf LDs and SEs contents, suggesting that potato leaf LDs contained SEs (Kopischke et al., 2013). LDs are also synthesized during senescence in plants; their neutral lipid cores include TAGs, SEs, and free fatty acids (Hudak and Thompson, 1997; Thompson et al., 1998a). The rubber bodies of the commercial rubber tree, *Hevea brasiliensis*, are similar to other cytosolic LDs, consisting of hydrophobic isoprenoid core (Siler et al., 1997; Cornish et al., 1999). Phloem exudates from stems of carnation and rapeseed contain LDs with a variety of neutral lipids including SEs, waxes, free fatty acids and TAGs (Thompson et al., 1998b). The LDs from chromoplasts contains TAGs and carotenoids (Deruere et al., 1994). In contrast, the plastoglobuli from chloroplasts is made of TAG and phytyl esters under nitrogen limiting growth conditions (Gaude et al., 2007). It’s believed that LDs play a role in keeping the free sterol content of cell membranes homeostasis (Schaller, 2004). It’s not surprising that free sterol biosynthesis also is subjected to strict regulation. Expression of *3-hydroxy-3-methylglutaryl-coenzyme A reductase 1* (*HMGR1*) in to tobacco resulted in sterol overproduction (Schaller et al., 1995), suggesting that HMGRs are the crucial check point for sterol biosynthesis. In contrast, the enhanced *AtAST1* expression resulted in decreased level of free sterols and increased level of SEs (Chen et al., 2007). Sequence analysis identified four HMGR isoforms from *Camellia sinensis* genome, and they were named as *CsHMGR1*, *CsHMGR2*, *CsHMGR3*, and *CsHMGR4*, respectively, sequence analysis also identified one of *AtAST1* from tea tree genome (*CsAST1*).

LDs are dynamic organelle and their sizes or numbers could be modulated by genetic or environmental factors (Tatenaka et al., 2019). Expression of lipodystrophy protein *SEIPIN1* promoted accumulation of large-sized LDs, while expression of *SEIPIN2* and *SEIPIN3* promoted small LDs (Cai et al., 2015); constitutive over-expression of *Small Rubber Particle protein* (*SRPs*) homolog in *Arabidopsis* resulted in increased numbers of large LDs in postgermination seedling, and loss-of-function mutant lines exhibited the opposite phenotypes (Kim et al., 2016). In *Arabidopsis* leaves the LDs abundance varied considerably during the diurnal cycle, the numbers of LDs were higher at the beginning of the day, while they became lower at the end of the day (Gidda et al., 2016). LDs synthesis was promoted by leaf senescence (Mckegney et al., 1995). So far it remains unexplored how the size, numbers and compositions of LDs changed, and how *PSAT1, HMGRs*, or *AST1* expressed during leaf maturation process.

Tea (*Camellia sinensis* L.) is a popular beverage and is consumed in many parts of the world, partly due to its unique taste and aroma quality. Previous studies have suggested that the number of osmiophilic particles from chloroplast (plastoglobules) were positively correlated with the aroma quality of made tea. However, the chemical composition of plastoglobuli from tea tree leaves remains elusive (Yan, 1980; Chen et al., 1992). It is generally assumed that cytosolic LDs are ubiquitously present in all cell types, and the aroma quality of made tea has been speculated to correlate with LDs. However, little research on LDs has been conducted in tea tree. In this study, we characterized the cytosolic LDs from *Camellia sinensis* cv. *Tieguanyin*, an elite cultivar to make Oolong tea, which showed better aroma quality compared with other types of tea such as green tea or black tea. The chemical composition of cytosolic LDs was elucidated and their dynamic changes during leaf expansion and diurnal cycle were quantified. Our data suggest that cytosolic LDs from tea tree leaves mainly serve as storage of sterols and triterpenoids in the form of esters, and are not major contributors to the aroma quality of made tea.

## Materials and Methods

### Plant Material

**FIGURE 1:**
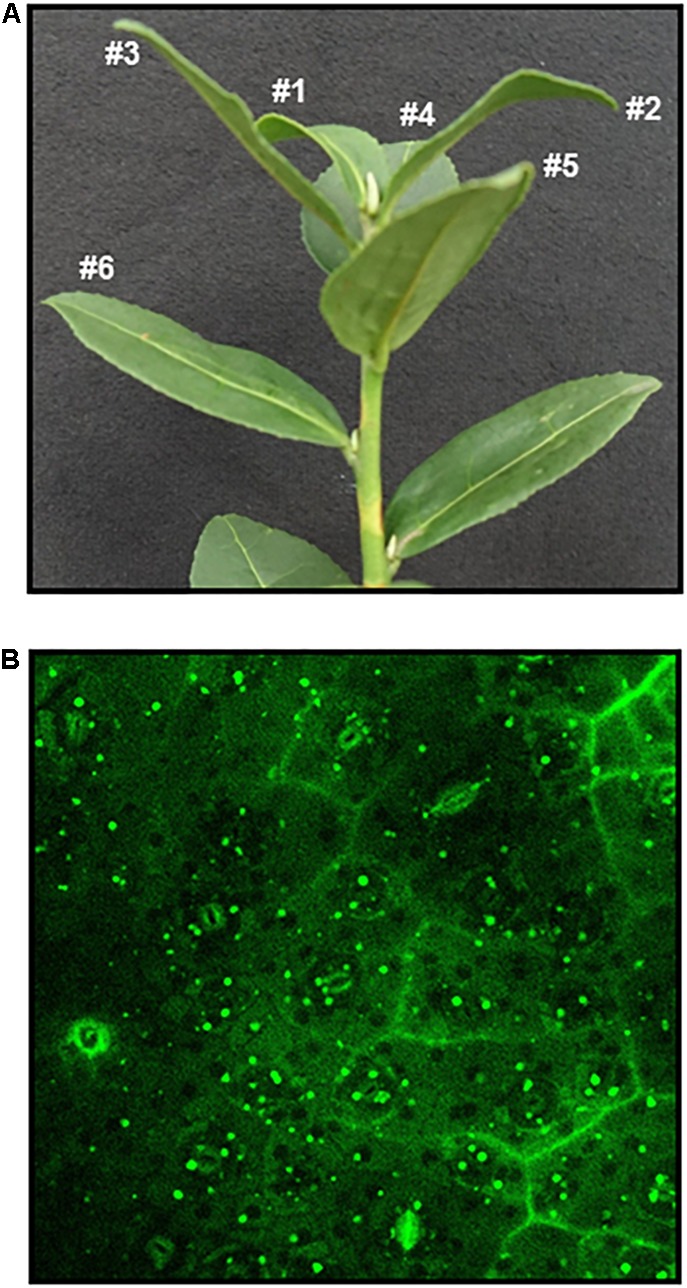
Nile Red staining of leaf lipid droplets from *Camellia sinensis* cv. *Tieguanyin*. The third leaf was harvested from a growing twig **(A)**, leaf discs were infiltrated in Nile Red solution (2 μg mL^-1^) in 50 mM PIPES buffer (pH 7) for 30 s, then stained for 30 min before observation under confocal microscopy, excitation was at 488 nm and emission was at 550–630 nm **(B)**. Bar = 20 μm.

*Camellia sinensis* cv. *Tieguanyin* was grown in a tea garden at Fujian Agriculture and Forestry University. Tea leaves were harvested after new shoots reached stage of one bud with six to seven leaves (Figure 1A).

### Imaging Lipid Droplets

Lipid droplets (LDs) were stained by either Nile Red or TopFluor cholesterol to visualize LDs *in situ*, then imaged by confocal scanning fluorescence microscopy (Zeiss LSM880, Jena, Germany). For Nile Red staining, leaf discs in 0.5 cm diameter were prepared from fresh leaves, transferred into a 20 mL syringe containing 5 mL Nile Red solution (2 μg mL^-1^, Solarbio, diluted from 1 mg mL^-1^ stock in DMSO) in 50 mM PIPES buffer (pH 7), infiltrated under negative pressure by pulling the plunger for 30 s, then returned to atmospheric pressure, and stained for additional 30 min before microscopic observation. For double staining with TopFluor-cholesterol and Nile Red, freshly prepared leaf discs were first infiltrated in 10 μmol L^-1^ TopFluor-cholesterol containing 30 μmol L^-1^ methyl-β-cyclodextrin (Avanti, United States, diluted from 30 mmol L^-1^ stock in ethanol), then stained in dark for 4 h (Hölttä-Vuori et al., 2008). The TopFluor-cholesterol staining solution was discarded, 5 mL Nile Red solution (2 μg mL^-1^) was added, and stained for additional 30 min before microscopic observation. For Nile Red imaging, excitation was at 488 nm and emission was at 550–630 nm (James et al., 2010; Gidda et al., 2016). For TopFluor-cholesterol imaging, the excitation was at 488 nm and emission was at 490–520 nm (Hölttä-Vuori et al., 2016). The Nile red signal was first captured, then change filter sets and take a picture of TopFluor-cholesterol signal. To count the LDs number, three shoots with same bud break time were harvested at the stage of 1 bud with 7 leaves, one leaf disc was prepared from each leaf position, then were stained in Nile Red solution for 30 min, the images were captured under confocal microscopy in Z-stack mode. LDs numbers were counted from each image and averaged by leaf area.

### Lipid Droplets Isolation

LDs isolation was following the methods described by Horn et al. (2013); Ding et al. (2013), and Niemann and Baas (1985a), with modification. Sixty gram of fresh tea leaves were weighted and transferred into a glass beaker, chloroform was added such that the leaves became fully submerged. After stirring for 30 s at room temperature, the chloroform was removed, then fresh chloroform was added, and the extraction was repeated. After cuticular wax removal, leaves were left in the hood for chloroform evaporation. The leaves were ground by mortar and pestle on ice with 160 mL of ice-cold buffer A (20 mM tricine, 250 mM sucrose (pH7.8), 0.2 mmol L^-1^ PMSF). The solution was transferred into 50 mL Eppendorf tube, and homogenized by polytron (IKA, Germany). The homogenization condition was: 14,400 rpm for 1 min, pause 30 s, repeat 4 times. The homogenized solution was centrifuged at 1,000 g for 1 min, the supernatant was passed through four layers of Miracloth. The filtered homogenate was collected into a fresh tube, centrifuged at 3,000 g for 10 min at 4°C. The supernatant was transferred into a new tube and 2 mL of buffer B (20 mM HEPES (pH 7.4), 100 mM KCl and 2 mM MgCl_2_) was added per 8–10 mL of supernatant, centrifuged at 4,000 g for 30 min at 4°C and the top phase transferred to a 1.5 mL centrifuge tube. The transferred material was centrifuged at 10,000 g for 10 min at 4°C and the LDs were carefully collected from the top, washed twice with 1 mL buffer B, and the pellet suspend in 1 mL buffer B.

### TLC Analysis

To isolate lipids from purified LDs, two volumes of chloroform: methanol (2:1, v/v) was added, vortex, then centrifuged at 13,600 g for 4 min, the lower phase was transferred into a new tube. Two volumes of chloroform: methanol (2:1, v/v) was added to the upper phase. This extraction was repeated once, and the lower phases were combined and dried down by CentriVap Console (Labconco, KS, United States). The pellet was dissolved in 30 μL chloroform and loaded onto a TLC plate and resolved with a mixture of hexane: diether ether: acetic acid (80:20:1, v/v/v). Lipids were visualized with iodine vapor.

### Terpenoid-Esters Isolation and Analysis

To measure terpenoid ester content changes with leaf growth, new shoots at stage of one bud with seven leaves were selected, the first leaf to the fifth leaf were separately harvested and pooled, four biological replicates were used for each leaf position. To quantify the diurnal changes of terpenoid esters, the third leaves from new shoots were harvested at 4 h intervals, the sample collection started at sunrise (5:00 AM), and ended at 1:00 AM the next day; sunset occurring at 5:00 PM. The third leaves were pooled and four biological replicates were used for each time point. Terpenoid ester isolation was conducted according to the method described by Wewer et al. (2011) and Wewer and Dörmann (2014). Fresh tea leaves (1.4 g) from *Camellia sinensis* cv. *Tieguanyin* were ground to fine power by mortar and pestle in liquid nitrogen. Total lipids were extracted with 20 mL of chloroform: methanol: formic acid (1:1:0.1, v/v/v) and 10 mL of 1 M KCl: 0.2 M H_3_PO_4_ solution. All organic solvents contained 0.01% butylated hydroxytoluene as antioxidant. 100 μg of 18:2-cholesterol was added as internal standard. The sample was vortexed and centrifuged (5,000 g, 5 min) to get phase separation. The lower, organic phase was transferred into a new tube. The upper phase was extracted twice with 10 mL of chloroform: methanol (2:1), the organic lower phase was combined. The solvent was evaporated under a stream of N_2_ gas. Nonpolar lipids were separated from polar lipids by CNWBOND Si SPE cartridge (2 g bed mass; ANPEL, Shanghai, China). Total lipids were dissolved in chloroform and applied to SPE column; nonpolar lipids (including free sterols and terpenoid esters) were eluted with 8 mL of chloroform, dried under a stream of N_2_ gas. The pellet was dissolved in 500 μL of hexane, then subject to an additional step of SPE separation. Terpenoid esters were eluted with 9 mL of hexane: diethylether (99:1, v/v), dried under a stream of N_2_ gas. Dried pellet was hydrolyzed in 1 mL of 6% (w/v) KOH in methanol at 90°C for 1 h, then extracted with equal volume of hexane, centrifuged at 100 g for 1 min. The upper phase was collected, then dried under a stream of N_2_ gas. Terpenoids were derivatized with 400 μL of N -methyl- N -(trimethylsilyl)-trifluoroacetamide (MSTFA) containing 1% TMCS at 80°C for 30 min, MSTFA was evaporated under an N_2_ gas stream, and the samples were dissolved in 600 μL hexane for GC analysis. Each sample was divided into two parts for GC-MS (GCMS-QP2010 Ultra, Shimadzu, Japan) and GC-FID (GC-2010 plus, Shimadzu, Japan) analysis, respectively. GC-MS and GC-FID were equipped with the same type of capillary GC column (DB-1, 30 m × 0.25 mm × 0.25 μm, Agilent, CA, United States). Oven temperature gradient was set as same for GC-MS and GC-FID: starting at 150°C, ramp 10°C min^-1^ to 280°C, keep constant for 10.5 min, then back to 150°C. Helium was used as mobile phase at speed 1.0 mL min^-1^, 1 μL of sample was injected at split ratio of 20:1.

### Gene Expression Analysis

Total RNA was isolated from the first leaf to the fifth leaf of the same shoot by using RNAprep Pure Plant Kit (Polysaccharides & Polyohenolics-rich) (Tiangen, Beijing, China). Two micro gram of total RNA from each leaf position was used to synthesize cDNA by M-MMLV reverse transcriptase. Quantitative real-time PCR was performed to monitor the expression levels of *CsPSAT1*, *CsHMGR1,2,3,4*, and *CsAST1* with SYBR green (Invitrogen, United States), *CsGAPDH* was used as internal control. The primer sequence and gene accession numbers were provided in Table 1. Fold difference was calculated using 2^-ΔΔCt^ method. Three biological replicates were performed and each sample analyzed by three technical replicates.

**Table 1:** The primer sequence for real time PCR.

Gene	Accession number	Primer sequence
*CsPSAT1*	MH194574	Forward: 5′-AAGCTAAGCGTGGACCTTTGAG-3′
		Reverse: 5′-AGCTCTGCCCAAGGTGAACA-3′
*CsHMGR1*	TEA019049	Forward: 5′-TAAACCTTCCCTCCCTGAA-3′
		Reverse:5′-GAGGAGGAGGAGGAGGACGA-3′
*CsHMGR2*	TEA017373	Forward:5′-AGACGTTGACCAGATTCTC-3′
		Reverse:5′-CGTCCGATGATTCAAAAGCT-3′
*CsHMGR3*	TEA002814	Forward:5′-CTCGAATTGGATGATGATGAT-3′
		Reverse:5′-ATTAGGGTCGCACGATAACA-3′
*CsHMGR4*	TEA028769	Forward:5′-GATTCTGACGACGGTGGAC-3′
		Reverse:5′-AACGGCAATCCATCTAGACT-3′
*CsAST1*	TEA001374	Forward:5′-AGAAAACCCAGCTCCCAAGTC-3′
		Reverse:5′-GCAGAAACAAACCCACAAG-3′
*CsGAPDH*	KA295375.1	Forward: 5′-TTTTTGGCCTTAGGAACCCAGAGG-3′
		Reverse: 5′-GGGCAGCAGCCTTATCCTTATCAGT-3′

## Results

### Cytosolic Lipid Droplets Changes With Leaf Expansion

To observe the presence of cytosolic LDs from tea tree leaves (*Camellia sinensis*), the third leaf from growing shoot was first stained by Nile Red, a widely used neutral lipid dye (Greenspan et al., 1985). Under confocal microscopy large amounts of cytosolic LDs were observed (Figure 1). Then the size and number of LDs from different leaf positions of a growing shoot were measured. We found that cytosolic LDs emerged from the first leaf, the number of LDs kept increasing from the second leaf to the fourth leaf, then decreased in the fifth leaf (Table 2). LDs size distribution from the first leaf to the fifth leaf showed dynamic changes, the first leaf was dominant by LDs with a diameter in the range of 0–3 μm, then the proportion of larger size LDs increased with leaf maturation. The small LDs (0–1 μm in diameter) were solely detected from the first and the second leaf position, and undetectable starting from the third leaf. In contrast, the proportion of larger size LDs (4–6 μm in diameter) increased from the first leaf to the fifth leaf (Table 2).

**Table 2:** The size distribution of lipid droplets from developing tea leaves.

Leaf position	LDs numbers/mm^2^	LDs size distribution (%)						
		0–1 μm	1–2 μm	2–3 μm	3–4 μm	4–5 μm	5–6 μm	6–7 μm
#1	319 ± 62	22.8 ± 3.2	39.5 ± 4.8	24.6 ± 3.2	9.7 ± 1.5	2.6 ± 0.4	0.9 ± 0.2	ND
#2	501 ± 91	16.0 ± 2.0	31.3 ± 4.3	35.1 ± 4.0	10.8 ± 1.7	6.3 ± 0.9	0.4 ± 0.1	ND
#3	804 ± 105	ND	10.3 ± 1.7	46.3 ± 5.3	35.2 ± 4.0	7.4 ± 1.0	0.7 ± 0.2	ND
#4	962 ± 112	ND	20.5 ± 2.5	41.4 ± 5.4	29.5 ± 3.9	7.2 ± 1.3	1.4 ± 0.3	ND
#5	511 ± 108	ND	16.4 ± 1.9	42.6 ± 4.9	26.6 ± 3.0	9.9 ± 1.9	2.7 ± 0.5	1.9 ± 0.4

*ND, not detected. Data was expressed as means ± standard deviation.*

### Terpenoid Esters and DAGs Were the Major Constituents of Leaf LDs From *Camellia sinensis* cv. *Tieguanyin*

**FIGURE 2:**
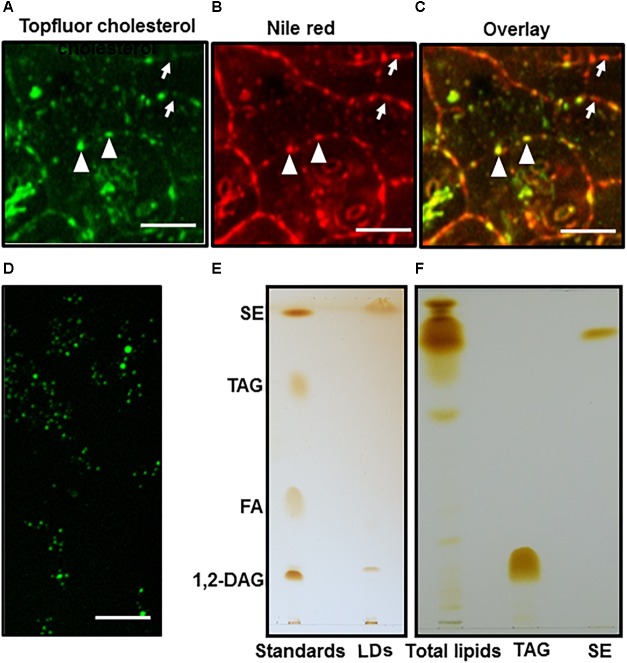
LDs imaging and TLC analysis. Fresh tea tree leaf discs were sequentially stained by TopFluor cholesterol and Nile red, then imaged for TopFluor cholesterol signal **(A)** and Nile Red signal **(B)**, images **(A,B)** were overlaid in **(C)**, bar = 10 μm. The LDs stained by Nile Red only were indicated by arrows, the LDs stained by both TopFluor cholesterol and Nile red were indicated triangle; **(D)** LDs were isolated from tea tree leaves, and stained by TopFluor cholesterol, bar = 20 μm; **(E)** Total lipid were isolated from purified LDs, then separated on TLC plate. The elution buffer was hexane: diether ether: acetic acid (80:20:1, v/v/v); **(F)** Total lipids were isolated from fresh tea leaves and analyzed by TLC. The elution buffer was hexane: diether ether: acetic acid (90:7.5:1, v/v/v). SE, sterol ester; TAG, triacylglycerol; FA, fatty acid; 1, 2-DAG, 1, 2-diacylglycerol.

The common neutral lipids of LDs in plants include TAG, DAG, and free fatty acids (Murphy, 2001). To observe if SEs coexist with other neutral lipids in *Camellia sinensis*, fresh tea leaves were sequentially stained with TopFluor cholesterol and Nile Red, then observed under confocal microscopy (Figure 2A–C). TopFluor cholesterol has been used previously to label LDs (Gocze and Freeman, 1994), and helped to elucidate sterol deposition within specific subcellular compartments (Hölttä-Vuori et al., 2016). The image overlay results indicated that cytosolic LDs from tea tree leaves can be divided into two groups: LDs that were stained by both TopFluor cholesterol and Nile Red (Figure 2A,B, indicated by triangles), and LDs that were stained by Nile Red only (Figure 2A,B, indicated by arrows). These observations suggest that LDs from tea leaves are heterogenous, and the neutral lipid composition could vary among individual LD.

To verify if LDs from tea leaves contain SEs, LDs were first isolated from tea leaves (Figure 2D), total lipids were isolated from purified LDs, and separated on TLC (Figure 2E). Based on the co-migration with lipid standards, two major components were identified from LDs: the most abundant one co-migrate with the SEs standard, followed by a band that co-migrates with 1, 2-DAG standard. In contrast, TAGs and FAs were detected in trace amounts (Figure 2E).

Previous reports demonstrated that TAGs are the major components of leaf LDs from other plant species (Parker and Murphy, 1981; Hudak and Thompson, 1997; Thompson et al., 1998b; Horn et al., 2011; Kopischke et al., 2013), our data from tea leaves suggests that the composition of LDs could be species-specific. Since TAGs can be easily converted to DAG by lipase, and the LDs isolation procedure involved tissue disintegration which could activate lipases, thus convert TAGs to DAGs. To test this possibility fresh tea leaves were immediately deactivated after harvesting by hot propanol, then total lipids were isolated and analyzed by TLC. TAGs were still detected in trace amount while an intense band co-migration with SE standard (Figure 2F). These observations excluded the possibility of TAGs conversion into DAGs during LDs isolation process, and support our conclusion that TAGs are minor components of tea leaf LDs.

Li et al. (2017) found that TAGs were prominent components in lipidomic study of black tea manufacturing processes, however, we found that only trace amounts of TAGs were detected from the cytosolic LDs of fresh leaves (Figure 2E). This difference suggested that large amounts of TAGs essentially were formed during post harvesting and processing of tea. This view was further supported by the increasing trend of TAGs during black tea processing (Li et al., 2017).

### Compositional Analysis of Sterol Esters From Fresh Tea Leaves

**FIGURE 3:**
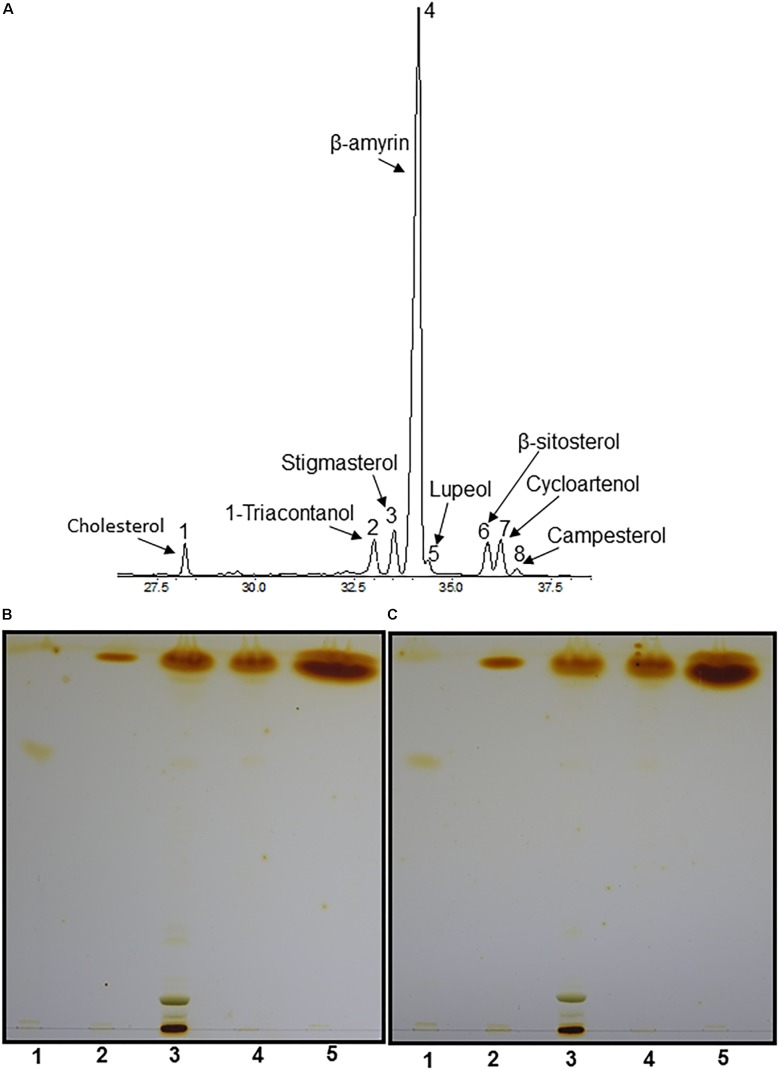
Compositional analyses of LDs by GC-MS. **(A)** GC-MS chromatography of terpenoid esters. Total lipids were isolated from 1.4 g fresh tea leaves, nonpolar lipids were separated by SPE column, then subjected to second SPE column to purify terpenoid esters. The purified terpenoid esters were hydrolyzed by 6% (w/v) KOH in methanol, terpenoids were extracted and derivatized with MSTFA/TMCS for GC-MS analysis. 1, Cholesterol; 2, 1-triacontanol; 3, stigmasterol; 4, β-amyrin; 5, lupeol; 6, β-sitosterol; 7, cycloartenol; 8, campesterol. **(B,C)** TLC analysis of terpenoid esters isolated from tea leaves without pre-wax removal and with pre-wax removal before total lipid isolation, the elution buffer was hexane: diether ether: acetic acid (80:20:1, v/v/v). Lane 1, β-amyrin standard; lane 2, sterol ester standard; lane 3, total lipids from tea leaves; lane 4, non-polar lipids isolated from total lipids; lane 5, terpenoid esters isolated from non-polar lipids.

To characterize the sterol composition of LDs, terpenoid esters were isolated from fresh tea leaves, then analyzed by GC-MS and GC-FID. Four different sterols were identified, including stigmasterol, β-sitosterol, cycloartenol and campesterol (Figure 3A). Sterol esters were reported to be present from black tea (Li et al., 2017), and we proposed that these SEs likely were derived from cytosolic LDs of fresh leaves. Unexpectedly, two triterpenoids (β-amyrin and lupeol) and one long chain alcohol (1-triacontanol) were also detected. Since before GC-MS analysis, terpenoid esters were first transesterified, raises the question whether detected β-amyrin was derived from β-amyrin ester or from contamination of free β-amyrin during terpenoid ester isolation. Free β-amyrin has been reported to present from tea leaf cuticular waxes (Zhu et al., 2018). To address this question, during terpenoid ester isolation partial samples were aliquoted from each step, including total lipids isolated from fresh tea leaves, non-polar lipids isolated from the total lipids after passing through the first SPE column, and the terpenoid esters isolated from the non-polar lipids after passing through the second SPE column. These samples were separated on TLC, free β-amyrin and sterol-ester standards also were included as control. As expected, free β-amyrin standard migrated slower than SE standard, likely due to the presence of hydroxyl group from free isoform which makes it more polar. Whether leaf waxes were removed or not prior to total leaf lipid isolation, trace amounts of free terpenoids, which co-migrate with free β-amyrin standard, were detected from total lipid isolate and the non-polar fraction (Figure 3B,C, lanes 3–4), but completely absent from the terpenoid ester fractions (Figure 3B,C, lane 5). These data demonstrated that the β-amyrin detected by GC-MS was indeed derived from β-amyrin esters instead of free β-amyrin contamination. Our data also suggested that triterpenoid esters probably co-migrate with sterol esters, due to their high structural similarity and hydrophobicity. Triterpenoid esters were also detected in leaf LDs of *Ilex aquifolium* L. (Niemann and Baas, 1985b), which also is a perennial evergreen shrub like the tea tree.

### SEs and Triterpenoid Esters Increased With Leaf Growth

**FIGURE 4:**
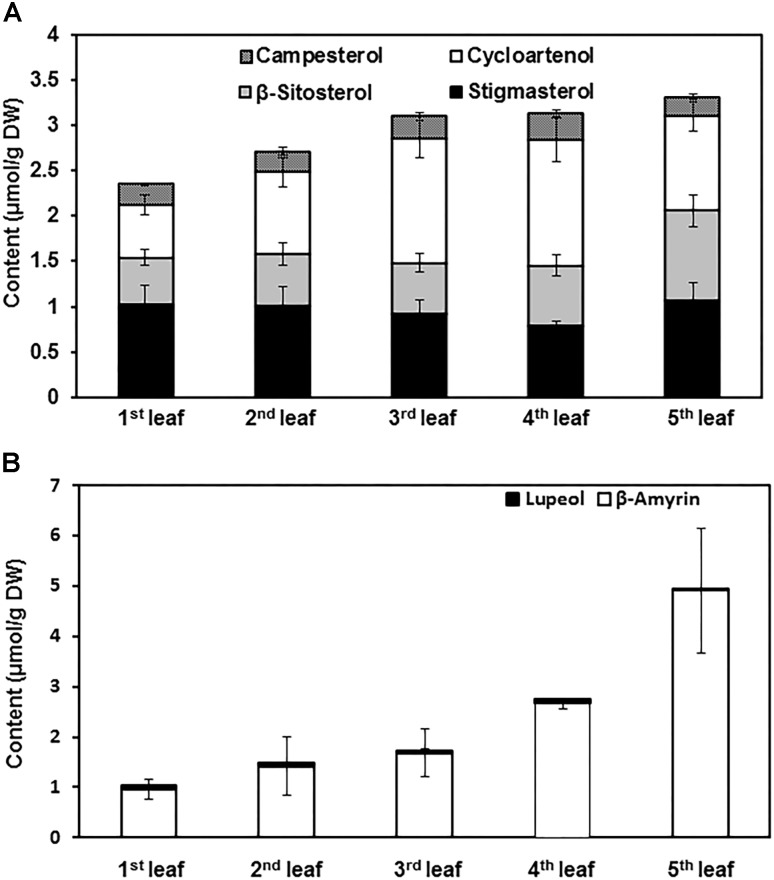
The contents of sterol esters and triterpenoid esters changed with leaf growth. **(A)** Sterol ester content changes from the first leaf to the fifth leaf. **(B)** Triterpenoid ester content changes from the first leaf to the fifth leaf. The first leaf to the fifth leaf was harvested from growing twigs, and pooled together by leaf position. Terpenoid esters were isolated and quantified from each leaf position. Four biological replicates were prepared; the data was expressed as average ± standard deviation based on dry leaf weight.

To quantify the changes of terpenoid esters with leaf expansion and maturation, terpenoid esters were isolated from the first leaf to the fifth leaf of growing shoots and quantified. Total contents of SEs increased with leaf position (Figure 4A). However, individual sterol esters showed different trends. Cycloartenol ester content increased from the first leaf to the forth leaf, then slightly decreased in the fifth leaf. In contrast, the contents of β-sitosterol ester and stigmasterol ester remained constant from the first leaf to the forth leaf, then increased in the fifth leaf. The content of campesterol ester remained constant regardless of leaf positions and maturation states (Figure 4A).

For the triterpenoid esters, β-amyrin esters were the most abundant components, only trace amount of lupeol esters were detected. The contents of β-amyrin esters increased stably from the first leaf to the fifth leaf. In contrast, lupeol ester contents only showed minor changes with leaf expansion and maturation (Figure 4B).

**FIGURE 5:**
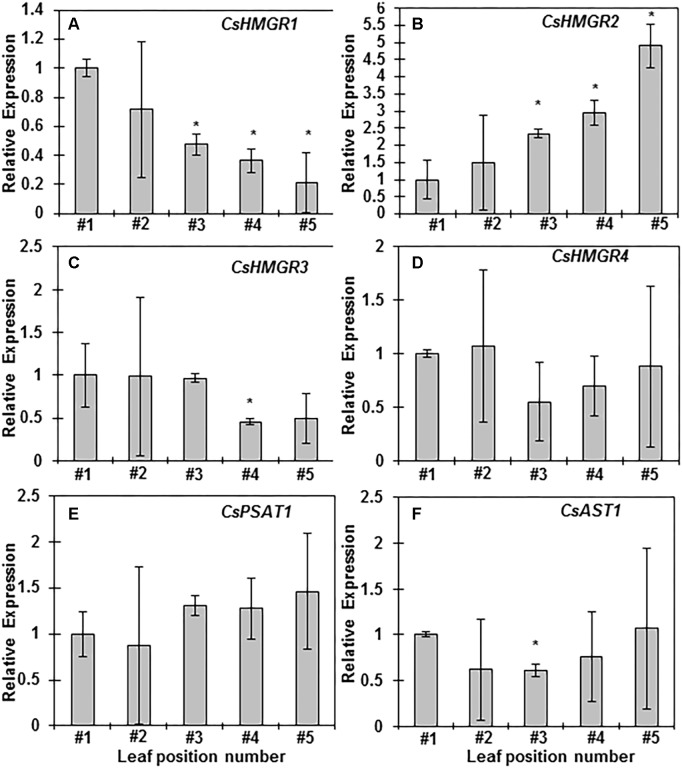
Sterol and sterol ester biosynthesis-related gene expression analyses during leaf maturation from growing shoots. **(A–F)** Relative gene expression levels of *CsHMGR1*, *CsHMGR2*, *CsHMGR3*, *CsHMGR4*, *CsPSAT1*, and *CsAST1* from the first leaf to the fifth leaf, respectively. Total RNAs were isolated from the first leaf to the fifth leaf, respectively. Q-PCR was conducted and 2^-ΔΔCt^ method was used to quantitate gene expression, *CsGAPDH* was used as reference gene, Three biological replicates were used for each leaf position. The data was expressed as average ± standard deviation. Statistical analysis was performed against the first leaf position, and significant change (*p* < 0.05) was labeled with asterisk.

To understand the molecular mechanisms underlining SEs changes during leaf maturation from a growing shoot, several genes involved in sterol or sterol ester biosynthesis were selected for quantitative gene expression analysis. HMGR is the rate-limiting step for terpenoid biosynthesis (Schaller et al., 1995). Sequence analysis identified four *HMGR* isoforms and they are named as *CsHMGR1*, *CsHMRG2*, *CsHMRG3*, and *CsHMRG4*, respectively. The expression of *CsHMGR1* reduced starting from the third leaf (Figure 5A); in contrast, the expression of *CsHMRG2* increased starting from the third leaf (Figure 5B). The expression of *CsHMRG3* and *CsHMRG4* were similar among different leaf position except the forth leaf showed reduced level of *CsHMRG3* (Figure 5C,D). The expression of *CsPSAT1* and *CsAST1* maintained constant with leaf maturation except the third leaf showed reduced expression of *CsAST1* (Figure 5E,F). Total sterol ester contents were correlated with the expression of *CsHMRG2*, suggesting that *CsHMRG2* is the key gene to determine terpenoid biosynthesis in tea tree leaves. Our data also suggested that *CsPSAT1* and *CsAST1* are not the rate-limiting step for SEs biosynthesis, instead the availability of free sterols determine how much SEs be synthesized.

### Diurnal Changes of Terpenoid Esters

**FIGURE 6:**
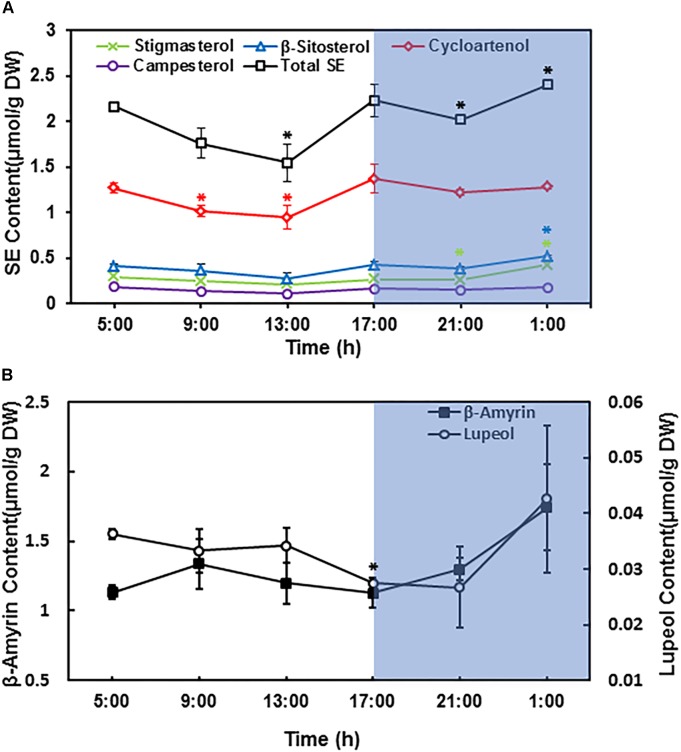
Sterol esters and triterpenoid esters changed during diurnal cycle. The third leaf from growing twig was harvested and pooled at 4 h interval, beginning at sunrise (5:00 AM). **(A)** Diurnal changes of leaf sterol ester contents. **(B)** Diurnal changes of leaf triterpenoid ester contents. Terpenoids esters were isolated and quantified from the third leaf position. Four biological replicates were prepared, and the data was expressed as average ± standard deviation based on dry leaf weight. Statistical analysis was performed against time point at 5:00 AM, and significant change (*p* < 0.05) was labeled with asterisk.

In *Arabidopsis* leaves the LDs abundance varied considerably during the diurnal cycle (Gidda et al., 2016). However, the number of LDs from tea leaves did not show clear diurnal changes as observed in *Arabidopsis*. Then we directly quantified the diurnal changes in terpenoid ester contents. The third leaf was harvested from growing shoot at 4 h interval during diurnal cycle, terpenoid esters were isolated and quantified. Upon illumination, individual SEs content started to decline, and reached the lowest peak right after noon; then started to increase and reached the highest peak at the end of the day. During night period, individual SEs showed different trends, while cycloartenol esters started to decrease, β-sitosterol esters and stigmasterol esters were stably higher. Campesterol esters remained constant during night period (Figure 6). Since cycloartenol is the upstream intermediate for β-sitosterol and stigmasterol synthesis, our observations suggest that LDs-stored cycloartenol are mobilized during the night for β-sitosterol and stigmasterol synthesis, the products could be stored in LDs in the form of esters and prepared for the usage upon light on.

The total SE contents showed similar trend as individual SEs during day time, however, during the night period total SEs decreased first followed by an increase; the increase in total SEs contents was mainly attributed by β-sitosterol esters and stigmasterol esters (Figure 6).

For triterpenoid esters, β-amyrin content was slightly increased after the first 4 h of lights on, and then gradually decreased until the end of day. During the night period, β-amyrin content started to increase again. In contrast, lupeol content only showed minor changes during diurnal cycle (Figure 6).

## Discussion

Previous studies suggested that the number of plastoglobules from fresh tea leaves was positively associated with the aroma quality of made tea (Yan, 1980; Chen et al., 1992), though the chemical composition of plastoglobules from tea leaves remains unknown. In *Arabidopsis*, plastoglobules are made of TAGs and phytyl esters (Gaude et al., 2007). Assuming that plastoglobules from tea leaves show a similar composition as *Arabidopsis*, the phytyl moiety could be released from its esters during tea processing, thus releasing the volatile aromatic phytyl. In addition, fatty acids released from TAGs also could be further oxidized during tea manufacture, and produce fatty acid-derived aroma compounds such as methyl jasmonate, cis-jasmone, hexanal, 3-hexenal, 3-hexen-1-ol (Hatanaka et al., 1974; Hatanaka et al., 1976; Mahanta et al., 1995; Dąbrowska and Boland, 2007; Matsui et al., 2017). However, TAGs might play minor roles for tea aroma production for two considerations: firstly, the majority of the liberated fatty acids during tea manufacture are attributed to autolysis of polar lipid classes in tea leaf tissue (Wright and Fishwick, 1979); secondly, the TAG content essentially increased during tea manufacture processes (Li et al., 2017).

In this study we found that cytosolic LDs of tea leaves only contained negligible TAGs and free fatty acids. In contrast, terpenoid esters were abundantly present in cytosolic LDs (Figure 2E) though their metabolic fate during tea processing is unclear. So far sterols and triterpenoids have not been reported as contributors for aroma generation. Thus, we speculate that cytosolic LDs from fresh tea leave play a minor role for tea aroma production. Based on its chemical compositon, cytosolic LDs could serve as a depot for triterpenoid ester and sterol ester storage in *Camellia sinensis*. Guo et al. (1995) reported that following seed germination, active sterol synthesis was initiated to provide building blocks for new membrane biogenesis. Similarly, on the rapid growing shoots, the tea leaf expands rapidly after bud break. It is expected that large amounts of sterols are demanded to support rapid leaf expansion, and part of sterols could be stored in the form of SEs to regulate leaf expansion. The steady increase of the total SEs pool with leaf expansion supports this view (Figure 4). We also observed that the contents of cycloartenol esters increased rapidly with leaf expansion and maturation, while other sterol esters only showed marginal changes (Figure 4A). Cycloartenol is the early common intermediate for the biosynthesis of stitosterol, stigmasterol, and campesterol. Since SEs pools were under rapid turnover with esterification and hydrolysis that occurred concomitantly (Dyas and Goad, 1993), the rapid increase of cycloartenol esters with leaf maturation suggests that cycloartenol esters serve as a ready to use pool for the synthesis of various sterols during leaf expansion stages.

LD abundance in *Arabidopsis* leaves showed diurnal change (Gidda et al., 2016). The number of LDs from tea leave did not show clear diurnal changes, instead the quantification data demonstrated that sterol esters showed diurnal changes (Figure 6). The compositional difference could account for this: The major component from *Arabidopsis* leave LDs is TAG (Gidda et al., 2016); in contrast, the tea LDs contains triterpene esters and sterol esters, both showed different diurnal cycle (Figure 6). It still remains unclear what’s the physiological relevance of the diurnal changes of sterol esters, this phenomenon may indirectly reflect the changes of membrane-bound free sterols, which have significant effects on membrane fluidity and permeability. It is anticipated that the physic-chemical properties of plasma membrane could be adjusted in response to light illumination.

It was unexpected that β-amyrin was the dominant constituent from terpenoid ester fraction of *Camellia sinensis* leaves (Figure 3). In *Arabidopsis*, AtPSAT1 showed low activity on β-amyrin (Banas et al., 2005). Sequence analysis demonstrated that CsPSAT1 shared 72% amino acid identity and 81.9% similarity with AtPSAT1, it remains an open question if CsPSAT1 could show higher activity on β-amyrin than its *Arabidopsis* homolog. In addition, triterpenoid biosynthesis is activated with leaf expansion (Zhu et al., 2018), more available free terpenoids also could stimulate ester formation even if CsPSAT1 showed low activity on β-amyrin. We found that plant cells keep the free triterpenoid at low level (Figure 3B,C). Storage free triterpenes in the form of esters inside cytosolic LDs can minimize the cell toxic effects of their free forms. Triterpenes were demonstrated to have antimicrobial-, antifeedant-, and insecticidal-activities (Osbourn, 1996; Fu et al., 2014; Yadav et al., 2014; Yang and Lin, 2017; Nzogong et al., 2018). Their accumulation during leaf growth in *Camellia sinensis* could serve as defense mechanism against pathogens and herbivores.

## Author Contributions

MC and CC conceived and designed research. XZ, XC, ZD, YZ, and XK conducted LDs isolation and lipid analysis. WZ and ZD performed RNA isolation and Q-PCR. XZ, XC, and YZ analyzed the data. MC wrote the manuscript. JT edited the manuscript. All authors read and approved the manuscript.

## Conflict of Interest Statement

The authors declare that the research was conducted in the absence of any commercial or financial relationships that could be construed as a potential conflict of interest.
